# Occurrence of Psychiatric Disorders, Self-Sufficiency Problems and Adverse Childhood Experiences in a Population Suspected of Violent Extremism

**DOI:** 10.3389/fpsyt.2022.779714

**Published:** 2022-02-15

**Authors:** Christel Grimbergen, Thijs Fassaert

**Affiliations:** ^1^Public Health Service, Amsterdam, Netherlands; ^2^Department of Child and Adolescent Psychiatry and Psychosocial Care, Amsterdam UMC, Amsterdam, Netherlands

**Keywords:** radicalization, violent extremism, terrorism, psychiatric disorders, mental health, self-sufficiency, public health, adverse childhood experiences

## Abstract

**Background:**

Public health-inspired programs for Countering Violent Extremism (CVE) have developed internationally in a relatively short period of time. Research into these programs is scarce. There is a need for information that helps drive public health interventions.

**Objectives:**

To present data on the occurrence of psychiatric disorders, self-sufficiency problems and adverse childhood experiences (ACE) in a population suspected of violent extremism.

**Methods:**

A cross-sectional study, with data from screening reports for 34 adult subjects included in a multi-agency case-based approach on violent extremism in Amsterdam, the Netherlands. Subjects were screened in the period between December 2015 to May 2021. Screening reports, which included the Screener for Intelligence and Learning Disabilities (SCIL) and the Dutch version of the Self-sufficiency Matrix (SSM-D), were used to gather information on the main outcome measures.

**Results:**

Major psychiatric disease categories were found to be mood and anxiety disorders and mild intellectual disability (each 29.4%), substance related disorders (35.3%), personality disorders (41.2%), and psychotic disorders (14.7%). Complex self-sufficiency problems, measured by the number of people who had self-sufficiency problems in 4+ domains and the number of people who had similar self-sufficiency problems as homeless people in Amsterdam, were found in 35.3 and 32.4% of the client sample. The most prevalent ACE were emotional neglect (47.1%), household mental illness (44.1%), and loss of a parent (38.2%), 35.3% had been exposed to 4+ ACE. An association was found between N_ACE_ and self-sufficiency problems on two domains, namely “Mental Health” (rho = 0.51, *p* = 0.002) and “Law and order” (rho = 0.42, *p* = 0.013).

**Conclusions:**

An accumulation of social and psychiatric problems in people suspected of violent extremism underlines the importance of professionals in health and social care being actively involved in developing CVE approaches.

## Introduction

It is generally assumed that law enforcement approaches alone are insufficient in countering violent extremism (CVE) and therefore need to be supplemented by public health approaches, thus shifting the focus to helping identify and engage with at-risk (vulnerable) individuals and intervene at earlier stages (1). Case-based or case-managed CVE programs have been developed internationally in a relatively short period of time, which in certain circumstances adopt a public health approach (2, 3). These combined law enforcement and public health (LEPH) approaches in violence prevention typically center around multi-agency collaborations in specific localities (4–6). “Local”, because in line with international strategy (7), municipalities and their security chains are often given joint responsibility for the early detection of potentially risky individuals (8). “Multi-agency”, because (mental) health and welfare authorities have important roles to play (1).

However, after more than a decade of developing such local CVE programs, research in the context of these programs is scarce and the question of “what works” is generally very difficult to answer (2, 9, 10). Current CVE approaches have an emerging, albeit small evidence-base in terms of risk and protective factors that drive public health interventions (3, 10). It has been argued that the scope and effectiveness of CVE programs is likely to be enhanced if research on violent extremism would be more in line with public health inspired violence research as well (3, 11). The social-ecological framework, for that matter, is a model that is used by organizations such as the World Health Organization and US Centers for Disease Control use in the development of public-health based violence prevention programs and in problem assessment and analysis (12). The framework is based on the notion that no single factor explains why some are at higher risk of interpersonal violence than others. Instead a number of interacting individual, social and environmental factors have to be taken into account the same argument has been made when it comes to explaining why individuals radicalize to violent extremism (13–15).

Against this background, the aim of this cross-sectional study is to present data on health (related) and social issues that have found to be prominent in violence prevention research. This is done in a population that consists of people suspected of violent extremism. Data were derived from a social-psychiatric health screening procedure conducted on a sample of clients who were participating in a CVE program in Amsterdam, in the Netherlands.

First and foremost, we present data on the prevalence of psychiatric issues among those enrolled in the program. This is important, given there has been a lack of studies that have drawn on data derived from clinical interviews or other associated primary sources involving individuals participating in CVE programs (3, 16). There is also the issue of differences in the measured prevalence of psychopathology depending upon the data sources that are used, with a higher prevalence in studies based on clinical examinations compared to studies that access police or judicial data or open sources (3, 10). The link between violent extremism, terrorism and psychiatric disorders has been the subject of a long-running debate that is still ongoing to date (17, 18). That is, various researchers have concluded that the prevalence of psychiatric disorders is not markedly elevated in groups of violent extremists and terrorists compared with the general population (13), which lead to the conclusion that psychopathology is irrelevant to understanding terrorism. On the other hand, these studies have been criticized in several ways (13, 18). Recent studies into certain subtypes of violent extremism (lone actor terrorists) in fact do find an increased prevalence of psychiatric disorders compared to the general population and other types of terrorists (13, 18–20). This includes schizophrenia, psychotic disorders, autistic spectrum disorders and anxiety disorders, which were more prevalent compared with the general population (15, 21). Suicide bombers may be more likely to have avoidant-dependent personality disorder, depressive and suicidal symptoms (22–25). Problematic personality traits were found in a group of Dutch Islamist extremists, with half of them displaying behavioral problems (26). Some studies have concluded that milder forms of psychopathology are relevant in some subtypes of terrorists, including group actors and leaders of terrorist cells (27). In general, these studies demonstrate that there is no common psychiatric diagnosis for violent extremists and terrorists: they are characterized by their diversity. Research into psychiatric disorders in radicalized populations (that is those not convicted of terrorist offenses) are relatively rare. In their systematic review, Trimbur et al. (28) found that the prevalence of mental disorders varied considerably from 6 to 41% across various studies. Only two studies included in this review (28) drew on clinical interviews and no study used standardized instruments to identify psychiatric disorders.

Second, this study addresses the occurrence of adverse childhood events (or experiences, referred to as ACE), such as physical abuse and parental loss in (early) childhood (29) in the same sample of radicalized individuals. That is, in spite of numerous studies in criminology that explain why and how exposure to ACE is associated with an increased risk of perpetrating violence and crime later in life, only few studies seem to have linked ACE to violent extremism (10, 30, 31). From a public health perspective on violence prevention, the concept of ACE is important for a number of reasons. That is, research into long-term outcomes of ACE has revealed graded dose-response relationships, indicating that as exposure to ACE increases, so does the likelihood and magnitude of a variety of negative outcomes in adulthood (31, 32). This includes psychiatric disorders (33, 34) and substance use disorders (SUD) (35). Additionally, it has been shown, that multi-problem young adults who face a combination of mental health problems, substance abuse and who had contacts with the courts, are likely to have experienced one or more ACEs during childhood (36). What is even more important, ACE can be prevented (37). Thus, studying which ACE are prevalent in people suspected of violent extremism may provide starting points for new preventive strategies. Finally, ACE are associated with an even wider range of negative outcomes, so much so that it has been suggested ACE have a non-specific damaging effect on a range of functions, behaviors and outcomes (32, 38). What is more, research has demonstrated that a wide range of life experiences, specific stressors and complex needs that are associated with mental health disorders can also be present in the life of lone-actor terrorists (39). In a recent systematic review (10) it was found that poor family and partner relations and experiences of social isolation were linked to radicalization. Unemployment was also found to be an important factor associated with extremism and in some studies extremists were found to be more often (chronically) homeless. Studies of lone-actors show the presence of recent important life events associated with loss, bereavement and a diminishing social standard prior to their violent extremist acts. Other complex needs that have been studied and have an association with radicalized individuals include traumatic experiences, for example physical, sexual, emotional abuse, parental abandonment, domestic violence and discrimination, both in early childhood and later in life (10). While these studies illustrate a range of complex needs, life experiences and stressors, they point to a highly individual spectrum of risk and protective factors (24, 40), which is in line with the aforementioned socio-ecological model (12). Against this background, the third and final set of outcomes in this study include self-sufficiency problems, defined as the (in)ability to realize an acceptable level of functioning, by organizing appropriate care or support in the most important domains of daily life (41). These domains include (but are not limited to) mental health and substance use, housing, finances and social relationships.

In sum, the main research questions for this study were (i) what is the prevalence of psychiatric issues, ACE and self-sufficiency problems amongst the sample and (ii) is there an association between (more) ACE and self-sufficiency problems? Regarding the latter, it was expected that we would find a small but statistically significant positive association.

## Methods

The Amsterdam CVE program from which our client sample is drawn is a multi-agency case-based approach which aims to limit security risks and prevent criminal offenses related to right-wing, left-wing, single issue and Islamist extremism. Primary target groups are (potential) terrorists, returnees and their recruiters or people who (plan to) travel to conflict areas. These people can be reported to the municipality or the police. Reports can come from various sources, including civilians (e.g., family members), professionals and organizations such as schools or employers. Once a person is reported to the program a quick scan is completed by professionals with a law enforcement background using data from police- and security service files. The quick-scan looks at evident high-risk behaviors (such as expressions that imply that subjects are influenced by extremist networks), suspicious criminal behaviors (such as incitement to violence, intimidation) and previous convictions for terrorism-related offenses (preparatory acts, membership of terrorist organizations). In addition, the nature of the risks and underlying problems are analyzed in a risk profile. Based on the outcomes of the quick-scan and risk profile, cases may be included in the approach. Enrollment in the program is not voluntary, in a sense that agencies are going to exchange personal information about cases and draw plans of action regardless of whether an individual participates. As a consequence, upon inclusion, all cases are appointed to a case manager, who may be connected to organizations such as the municipality, police and probation office. He or she is responsible for designing the plan of action. Drawing from recent insights (14, 15, 42), this plan includes measures in the domain of both justice and health care. Still, it is possible that individuals are enrolled and choose not to cooperate. Strictly speaking, there are no legal consequences, unless (for example) non-cooperation implies violation of parole conditions.

Since the provision of health care is an important feature of the program, the municipality's public health service (PHS) helps to identify clients with possible (mental) health and self-sufficiency problems. The goal is to assess individual needs, in order to help inform the personal plan of action (8). For that purpose, the PHS undertakes a brief social-psychiatric screening. Nowadays, screenings are offered to every subject included in the program. While enrollment in the CVE program is not voluntary, the screening is. Results of similar screenings by the PHS in other, forensic-type, populations have been published elsewhere (43–45). Screenings are preferably done at the main office of the PHS, but some clients are screened at other places such as in their home or in prison. The screening is a semi-structured interview that takes around 90–120 min and is conducted by a psychiatrist (the 1st author of this study) and a psychologist. Afterwards, the results are discussed with representatives of health care providers in Amsterdam. Together, they come up with an appropriate plan of action, which may or may not include a medical referral.

Unfortunately, an elaborate psychiatric evaluation [i.e., with an analysis of all possible causes, course, and consequences for the interviewee (46)] is not feasible. This is due to the extensive list of topics that need addressing, combined with time constraints. The only structured questionnaire that is administered is a screening tool for mild to borderline intellectual disability (see the description of the measures below). The semi-structured interview is guided by a topic list and structured around the same essential life-domains that are included in the Dutch version of the Self-sufficiency Matrix (SSM-D) (41, 47). These themes include (but are not limited to) physical and mental health, addiction, housing, income and social relations. See “measures” for more information on the SSM-D. The interviewer uses standard wording of the initial questions to be asked (e.g., “Do you have any physical symptoms/complaints? To what extent do physical symptoms impair the performance of your activities in daily life?”). If answers are positive and/or need more clarification, the interviewer continues with a freeform enquiry. The order in which topics are discussed and questions are asked is not fixed (semi-structured) and applied according to the course of the conversation with the subject. It is important to know that the PHS is not aware of the reason why people are referred to/included in the program, nor does the PHS know if or when an individual exits the program. This (judicial) information is not provided to the PHS, nor is it the purpose of the screening to measure it. Some subjects chose to disclose this information, however because of the self-report nature of the data used in this study we have decided not to include this information here. Hence, this judicial information is outside the scope of this study.

The semi-structured interview scheme includes a brief but systematic evaluation of psychiatric symptoms which are reported or observed at the time of the interview. Based on this evaluation a provisional working diagnosis is defined. In line with Dutch professional guidelines (48), symptoms were divided into: cognitive functions (e.g., consciousness, attention, and concentration), affective functions (e.g., mood and affect), conative functions (psychomotor activities, motivation and behavior) and a description of personality trades and symptoms.

The quality of the entire interview in general, and the psychiatric assessment in particular, is warranted by the involvement of a psychiatrist and requires continuous professional education and training. Besides that there is an involvement of representatives of external health care providers with whom the results are shared and discussed. They also compare the outcomes to information in their electronic files, provided that consent is given by the client. If the latter is the case (i.e., patient consents), than those health care providers are also asked to provide additional medical information. For example previous forensic psychiatric evaluations or psychiatric assessments.

People may enter and later exit the program. From December 2015 to May 2021, a total number of 109 individuals had been enrolled in the program, of whom 38 people have been screened. Only those who consented to the screening and who were an adult (i.e., 18 years and older) at the time of screening were included in the study sample (*N* = 34).

### Measures

The primary outcome measure was a preliminary diagnosis for psychiatric morbidity. This was based on the working diagnoses, including differential diagnoses, categorized according to the main categories of DSM-IV (49) or DSM-V (50). A differential diagnosis was defined as one or more possible conditions or disorders that could be causing the symptoms in question. As such, differential diagnoses were applied when the presence of a disorder was suspected on the basis of its symptoms, but a more thorough assessment remained necessary to ascertain its presence.

A screening tool for mild to borderline intellectual disability (MBID), namely the Screener for Intelligence and Learning Disabilities (SCIL) (51) is the only validated screening tool used in the interview. It is included as a fast way to estimate a client's intellectual capacities, which may be helpful in the provision of adapted treatment in places or situations where this would not otherwise occur. The SCIL was developed as a tool to screen for MBID (i.e., IQ <85) among forensic populations (52, 53). Administration time is ~10 min and should be done by mental health professionals, although no specific training or education is required (51). The SCIL consists of 14 items (each scored with 0–2 points) in four domains: education, social contacts, school skills and language comprehension. This includes questions about education level and brief reading, writing and calculation tasks (53). The total score thus varies from 0 to 28 points. Previous studies have found good internal consistency and predictive accuracy (51, 53).

Presence of self-sufficiency problems (SSP) was measured using the Dutch version of the Self-Sufficiency Matrix (SSM-D). Self-sufficiency matrices were first developed in the U.S. (47). In 2010, the Self-Sufficiency Matrix was introduced in the Netherlands and developed as an observational screening tool that provides a reliable assessment of the degree of self-sufficiency in 11 essential life domains such as daytime activities, physical health, mental health, finances, domestic relations and daily life skills (41, 54). Each SSM-D domain is measured on a 5-point scale with 1 = “acute problems;” 2 = “not self-sufficient;” 3 = “barely self-sufficient;” 4 = “adequately self-sufficient;” and 5 = “completely self-sufficient”. Table 1 contains a brief description of each SSM-D domain. The SSM-D has adequate psychometric properties, including a solid single factor structure (i.e., self-sufficiency) and good internal consistency (Cronbach's alpha 0.85–0.89). In addition, Fassaert et al. (47) showed strong and statistically significant correlations between the SSM-D and well-known, extensively validated instruments like the Health of the Nation Outcome Scales (HoNOS) (55) and the Camberwell assessment of need short appraisal schedule (CANSAS) (56). Scores on each SSM-D domain were dichotomized (scores <3 vs. scores ≥3). Additionally, individual SSM-D scores were used to identify which proportion of the study population had severe (complex) self-sufficiency problems. This was done in two ways, namely by calculating (i) the number of people who had self-sufficiency problems in multiple domains and (ii) the number of people who had a combination of self-sufficiency problems that is typical for homeless people in Amsterdam. The latter was done similar to Fassaert et al. (43) and Buster et al. (57), who determined this based on scores <3 in the SSM-D domains of “Mental Health” or “Substances”, combined with scores <3 in either “Finances,” “Work and education,” or “Housing”.

**Table 1:** Description of SSM-D domains.

**Domain**	**Description**
Finances	Refers to the degree to which a person has at least sufficient income to cover their basic needs, that they obtain this income as independently as possible and that the income keeps pace with spending.
Work and education	Refers to being in paid work, being on a program to work (aimed at occupational participation or reintegration) or taking a course. When no paid work is being done, the activities being undertaken to find work are important.
Time use	Refers to the extent to which activities during the day are experienced as being pleasurable or useful by the person, the degree to which a person structures their day and the person's day-night rhythm.
Housing	Refers to the stability, quality and autonomy of the person's living conditions. The central question here is whether the person has a safe, adequate home where they can stay for an extended period
Domestic relations	Refers to the question of whether the person has good relationships with the people with whom he or she shares a household.
Mental health	Refers to the presence or absence of mental health problems and, if mental health problems are present, how the person copes with them.
Physical health	Refers to the presence or absence of a physical disorder and – if present – how the person copes with it.
Substance use	Refers to a person's use of drugs, gambling and use of alcohol and the effect it has on the person's day-to-day functioning.
Basic activities daily life (ADL)	Refers to the extent to which the person carries out and has carried out those activities that he or she needs to do to maintain their physical safety and welfare.
Instrumental ADL	Refers to carrying out activities—and the quality of their execution—which a person does to function safely and sustainably in their environment.
Social network	Refers to the number and quality of relationships with friends, family and acquaintances (who do not form part of the household).
Community participation	Refers to the degree to which the person participates in structured community activities and organizations.
Law and order	Refers to whether the person is currently (or has recently been) involved with the police and the law.

Finally, we gathered information from the semi-structured interview on exposure to ACE. This was done in a similar way as Segeren et al., (44) who originally used items from the Juvenile Forensic Profile (FPJ) (58, 59) to score ACE in archived youth-care files. The FPJ consists of 70 items and was developed to measure criminogenic risk factors in patient files. For this study, we selected the same items as Segeren et al. (44) used, namely the items that corresponded with the major ACE according to the CDC-Kaiser Permanente ACE Study (29). These are abuse, physical neglect, emotional neglect, sexual abuse, incarceration of a family member, household substance abuse, household mental health problems, household partner violence and loss of a parent. (See Table 2) for the scoring protocol. The FPJ does not distinguish between physical and emotional abuse, unlike the ACE construct most commonly used (60), so that one composite variable (i.e., Abuse) was scored. Occurrence of the ACE “Incarceration of a family member” was indicated in cases where one or more family members were convicted of a criminal offense. Emotional neglect was scored positively (yes) in cases where for example there was temporary/permanent separation from a parent or ignoring by/mental absence of a parent. Missing scores on all items were recoded to 0 (no). A cumulative ACE score was then calculated that ranged from 0 (no ACE exposure) to 9 (exposed to all ACE categories included in the study).

**Table 2:** Scoring protocol for the presence of ACEs based on FPJ-items.

**ACE**	**FPJ items**	**Score/category**
Abuse	Abuse by parents (FPJ)	Moderate/severe
	Abuse by others (FPJ)	
Physical neglect	Neglect (FPJ)	Moderate/severe
Emotional neglect	Unavailability of parents (FPJ)	Severe
Sexual abuse	Sexual abuse by parents (FPJ)	Moderate/severe
	Sexual abuse by others (FPJ)	
Incarceration family member	Criminal family members (FPJ)	Severe
Household substance abuse	Parental substance abuse (FPJ)	Moderate/severe
Household mental health problems	Parental mental health problems (FPJ)	Moderate/severe
Household partner violence	Domestic violence (FPJ)	Moderate/severe
Loss of a parent	Death of a parent	Yes
	Parental divorce (witnessed at age 4 or older)	Yes

#### Ethics Approval and Consent to Participate

All data are routinely collected and used for clinical practice. Subjects are not treated according to a particular study protocol and participation in the interview occurs on a voluntary basis. The Dutch law on Medical Research allows the use of this type of data for purposes of scientific research without an explicit informed consent, provided that the privacy of patients is fully ensured. The latter was achieved by the application of encoded patient numbers and reporting of results on adequately aggregated levels. As a result, the Medical Ethics Review Committee of the Academic Medical Center Amsterdam granted a “waiver of consent” for this study (W21_189 # 21.205), meaning that the Medical Research Involving Human Subjects Act (WMO) does not apply to this study.

#### Analysis

All analyses were done in SPSS version 21 (61). Descriptive analyses provided the sociodemographic information of the sample, and prevalence of psychiatric disorders, self-sufficiency problems and ACE, respectively. Finally, due to the limited sample size, the association between the total number of ACE (cumulative per person) and SSP (yes/no) was explored using only simple correlation coefficients (i.e., Spearman's rho).

## Results

In this sample of 34 subjects, 29 (85.3%) were male. Their mean age at time of screening was 27.6 years (sd. 5.5, range = 19–39). Most subjects (*N* = 30; 88.2%) were single, of whom five were either widowed or divorced. Thirty subjects (88.2%) had a migrant background, 22 of whom could be classified as a 2nd generation migrant. This means that they were born in the Netherlands but had at least one parent who was born abroad, in this case Morocco and Turkey. First generation migrants were born abroad themselves, namely in Morocco, Surinam, Turkey, Syria and Egypt.

Table 3 presents prevalence estimates in relation to psychiatric disease categories. Major psychiatric disease categories were mood and anxiety disorders and mild intellectual disability (each 29.4%), substance related disorders (35.3%), personality disorders (41.2%), and psychotic disorders (14.7%). In only four cases no indications for a mental health problem were found, while 23.5% of the sample had indications for a possible disorder in one category and 64.7% in at least two separate categories.

**Table 3:** Prevalence of possible psychiatric disorders in subjects (*N* = 34) included in a case-based approach of radicalization in Amsterdam (January 2015–present).

**Psychiatric disorder categories^**a**^**	**Prevalence (%)**
Psychotic	14.7
Mood/anxiety	29.4
PTSD	17.6
Hyperactivity/impulse control	5.9
Substance related^b^	35.3
Autism spectrum	11.8
Other axis 1^c^	23.5
Personality disorder	41.2
(Mild) intellectual disability^d^	29.4
Traumatic brain injury^e^	8.8

a *The working diagnosis consists of classifications made by (previous) mental health professionals and diagnoses from the screening. The table presents the prevalence including both previous and present classifications (higher bound number), even though some classifications have to be confirmed after referral*.

b *Mainly cannabis, alcohol, and cocaine*.

c *Includes attachment and/or identity disorders, sleeping disorders, dyslexia*.

d *Measured with the SCIL*.

e *Data available for N = 28*.

Table 4 presents on which life domains subjects experience self-sufficiency problems, in descending order. Self-sufficiency problems in work and education were most prevalent, which according to the SSM-D means that 47.1% of all subjects had no or inadequate income for basic needs *or* spontaneous or inappropriate spending, in combination with increasing financial debts. Second, 41.2% had problems in the domain of “Law and Order”, suggesting contacts with police at least frequently (several times a year) *or* pending law cases. Problems in “Time use” indicated that 38.7% had no or very little pleasurable/ useful activities, no or very little structure in day-to-day activities and/or an abnormal day/-night routine. Regarding “Community participation, 32.4% lived isolated from the community *or* caused some form of nuisance. In terms of “Social network”, a similar proportion had no or very little contact with family, no or very few pro-social contacts and many/solely negative social contacts. Another 32.4% reported problems in “Instrumental ADL”, which suggested one or more activities (e.g., cooking, medication management, taking care of administration, and other paperwork) were not carried out or there were limitations in several areas. Problems in this domain included signs of home pollution (e.g., a messy household) and under-/overmedication. “Mental health” was a problematic area in 29.4%, which suggested that there was an untreated (recurrent) mental illness present, as a consequence of which the person's functioning was severely impaired. Self-sufficiency problems in “Finances” were prevalent in 26.5%, which implicates inadequate income for basic needs *or* spontaneous or inappropriate spending and increasing debts. The same number of people reported problems in terms of “Housing”, which pointed at housing that was not suited for permanent habitation, situations in which rent/mortgage payment was not affordable, imminent threat of eviction or even homelessness. Finally, “Substance use” was a problematic area for 23.5%, which suggested the presence of a substance abuse disorder (addiction) which caused/worsened physical/mental health problems, while treatment was typically absent. Only a few people reported self-sufficiency problems regarding “Basic ADL” (daily activities related to personal self-care like hygiene, clean clothing), “Domestic relations” and “Physical health”.

**Table 4:** Self-sufficiency problems^a^ in subjects (*N* = 34) included in a case-based approach of radicalization in Amsterdam (January 2015–present).

**Domain**	**Self-sufficiency problems (%)^**b**^**	**No self-sufficiency problems (%)^**c**^**
Work and education	47.1	52.9
Law and order	41.2	58.8
Time use (*N* = 31)	38.7	61.3
Community participation	32.4	67.6
Social network	32.4	67.6
Instrumental ADL	32.4	67.6
Mental health	29.4	70.6
Finances	26.5	73.5
Housing	26.5	73.5
Substance use	23.5	76.5
Basic activities daily life (ADL)	5.9	94.1
Domestic relations	5.9	94.1
Physical health	0.0	100.0
**Number of SSM-D domains with problems**	%	
0	26.5	
1–3	38.2	
4+	35.3	
**Severe (complex) self-sufficiency problems** ^**d**^	32.4	

a *Measured with the Dutch version of the Self-sufficiency Matrix (SSM-D) (21, 37)*.

b *Acute problems, not self-sufficient (scores 1–2)*.

c *Barely, adequately, or completely self-sufficient (scores 3–5)*.

d *Based on scores <3 in the SSM-D domains of “Mental Health” or “Substances”, combined with scores <3 in either “Finances,” “Work and education,” or “Housing” (33, 46)*.

Complex self-sufficiency problems, measured by the number of people who had self-sufficiency problems in 4+ domains, were found in 35.3%. Additionally, 32.4% had a combination of self-sufficiency problems that is typical for homeless people in Amsterdam. Taken together, only nine subjects (26.5%) were at least barely self-sufficient on all SSM-D domains.

Of the total sample, 70.6% reported at least one ACE. The most prevalent ACEs (Table 5) were emotional neglect (47.1%), household mental illness (44.1%) and loss of a parent (38.2%). Sexual abuse and physical neglect were mentioned least often. On average, subjects had been exposed to 2.1 different ACE types and 35.3% had been exposed to 4+ ACE (Table 4). Finally, there was a statistically significant positive association between the number of ACE and self-sufficiency problems (SSP, results not in table), however only with respect to two domains, namely “Mental Health” (rho = 0.51, *p* = 0.002) and “Law and order” (rho = 0.42, *p* = 0.013).

**Table 5:** Prevalence estimates of distinct childhood adverse experiences and mean ACE score in subjects (*N* = 34) included in a case-based approach of radicalization in Amsterdam (January 2015–present).

**ACEs**	**Prevalence (%)**
Emotional neglect	47.1
Household mental illness	44.1
Loss of a parent	38.2
Household partner violence	23.5
Abuse	23.5
Incarceration family member	17.6
Household substance abuse	17.6
Sexual abuse	2.9
Physical neglect	0.0
**Number of ACEs**	**%**
0	29.4
1-3	35.3
4+	35.3
ACE mean (SD)	2.1 (2.0)

## Discussion

This study presents cross-sectional data on the occurrence of psychiatric disorders and additional self-sufficiency problems in a sample of Amsterdam citizens who are enrolled in a multi-agency case-based approach to address violent extremism and who are typically not convicted for extremist acts. Data were available for a small group of individuals, who were relatively young, predominantly male and single. Most of them had a migrant background, meaning that either they or (one of) their parents was born abroad, in countries such as Morocco, Turkey, Syria or Egypt. This profile is not very different from populations in other studies (3). It furthermore shows that psychiatric disorders are relatively frequently found in this group, which appears to be in line with previous findings in this field as well (10, 19, 21, 26). Compared to other research done in populations convicted of terrorist acts (10, 13) the percentages of mental disorders in our study are higher, especially for mild intellectual disability, personality and addiction disorders. For that matter, prevalence rates for psychiatric disorders appear to be more in line with research done in radicalized populations, in which percentages vary considerably between studies (28). Moreover, the sample presented with a considerable number of self-sufficiency problems on various life domains, with “Work and education” and “Social network” among some of the highest ranking problem areas, which is also consistent with previous findings on the complex needs of terrorist populations (10). Finally, ACE were generally highly prevalent and this is line with research on terrorist groups (10) and radicalized individuals and groups (28). In fact, in more than one third of this group (35.3%) we found indications for the presence of at least four ACE, which is a widely used cut-point to define “high risk” status for a myriad of adverse life outcomes (62).

A strength of this study is the availability of clinical data from a unique sample of radicalized individuals, since most empirical research has focused on subjects who were convicted of terrorism charges or who had been killed in the act of terrorism (13, 15, 20). So far only few studies based on psychiatric assessment of a radicalized population have been published (28). There are several reasons why health research in this area is difficult and why its results remain controversial, particularly regarding the prevalence of psychopathology (14, 15, 19, 20, 26). For example, violent extremism is a complex phenomenon with many different variables contributing to the process. In addition, there is a strong influence of the local political and societal context in which studies are conducted, which limits comparison across various types of extremism and across jurisdictions that can use different screening and risk assessment tools. Another strength of this study is the combination of clinical data on psychiatric morbidity with self-sufficiency problems and ACE, which together provides quite a broad perspective on the functioning of and complex needs within this specific population. To the best of our knowledge, this combination of information is relatively new and useful in thinking about developing individualized (treatment) interventions in CVE programs.

A number of limitations of the study should be considered as well. First, data were obtained from a screening procedure in a unique, but relatively small and specific sample, which raises questions about the generalizability of the results. Selection bias should be considered, for example because not all people included in the program were screened by the PHS. That is, nowadays the screening is offered to every subject in the program, but in the early years of the program this used to be a decision of the individual case-manager. Also, people who successfully travel to conflict zones or plainly refuse to participate in screening can be argued to have less social and/or mental health problems. So the individuals who were not able to be screened, might have less psychopathology and self-sufficiency problems. However, in his study of 140 Dutch Islamist extremists–which also consisted of individuals who traveled to conflict zones- Weenink (26) found indications for psychological and social problems in 60% of the sample. An additional limitation is that this is a cross-sectional study, which contains purely descriptive results. Thus, it is impossible to address the issue of causal relationships between ACE, psychiatric symptoms, and self-sufficiency problems. Finally, it is important to note that the screening mainly results in self-report data. Fortunately, in some cases, subjects provide consent to retrieve additional medical information from previous forensic psychiatric evaluations or psychiatric assessments. Nevertheless, self-report data have limitations. For example, due to the lack of more extensive information about personal histories, it was difficult to determine ACE in this sample. As a result, we have probably been able to determine ACE less accurately. The same counts for judicial data; we did not have reliable information about the radicalized and criminal acts of the individuals, so we did not include this information in the current study. This is unfortunate, because although mental health problems are relatively common in these groups and are more easily identifiable in studies which rely on clinical examinations (10), the body of literature studying the nature of the link between mental illness and violent extremism and terrorism is less well developed and hampered by methodological limitations (14, 15). Recent studies have focused on the possible functional links between individual aspects of psychiatric disorders and violent extremism or terrorism in order to fill this gap. This information can be deducted by case or vignette studies and may be especially relevant in mental disorders like autism spectrum disorders, psychosis, PTSD and addictions (10, 31, 63, 64). Further research is needed to shed more light on the role of mental disorder in the complex, individualized, heterogenous pathways to radicalization and terrorism (10, 14, 40, 63). In the context of the screening for the CVE program, however, it is important to note that we actually do formulate individualized hypotheses on the association between the problem areas that are observed during the screening and the process of radicalization or violent behavior. If relevant, this hypothesis is then included in the referral to the mental health organization, so that it can be included in the forensic analysis that forms the base of a treatment intervention.

Self-sufficiency problems were frequently present. A considerable number of individuals appeared to be vulnerable in several ways. In fact, we found that 32.4% of CVE subjects had severe and complex problems to an extent that is comparable with homeless clients in Amsterdam (40). This finding is similar to a previous study by Fassaert et al. (43) in a population of offenders of serious violent crime, of whom 35.9% had complex self-sufficiency problems to an extent that was comparable to homeless people in Amsterdam. That particular study was done in the context of a diversion program for violent repeat offenders in Amsterdam (the “Top600” approach) (65) which, in terms of organization and interventions, is comparable to the Amsterdam CVE program. Thus, once included in the program for violent repeat offenders, individual offenders are screened in similar ways by the PHS as are individuals suspected of violent extremism. However, while both target populations hold similar proportions of vulnerable individuals, the problem areas slightly differ in terms of ranking. For example, Segeren et al. (44) found that 54.7% of violent offenders had self-sufficiency problems with respect to Finances, in contrast to 26.5% in the current study population. Finally, a striking result was that “mental health” was a problematic area in about one-third of the study population, which might be considered lower than expected based on the prevalence of suspected psychiatric disorders in the present study population (i.e., indications for a possible psychiatric disorder in ~90% of the sample in the current study). However, it is important to bear in mind that, apart from the clinical assessment, the estimated prevalence of psychiatric disorders in the present study was also based on information from personal medical histories, whereas self-sufficiency is determined only on the basis of a person's current functioning. What is more, from the way the concept of self-sufficiency is applied in the SSM-D, it follows that a person with a disorder who receives some treatment or professional support is more self-sufficient than someone with the same medical condition who does not (adhere to) medical treatment. This is what distinguishes the SSM-D from instruments that solely focus on presence of symptoms or problems.

In more than one third of this group we found indications for presence of at least four ACE, which is often used as a red flag indicator for a high-risk status regarding a myriad of outcomes (62). What is more, there were some indications that occurrence of more ACE was associated with self-sufficiency problems, for example in relation to mental health. This finding concurs with the Segeren et al. study (44) among violent offenders and generally supports the notion that increased exposure to childhood trauma is associated with various negative outcomes in (young) adult life. Considering that the research exploring links between trauma and violent extremism is still in its infancy and the causal role of trauma in radicalization remains unclear (66–68), we believe the current results encourage further exploration of this topic.

As a result of the descriptive nature of this study and the small sample size, we cannot be sure if mental health, adverse child experiences and self-sufficiency problems are relevant to violent extremism. Still, we know for sure they exist in this sample, as they do in other groups in Amsterdam that come into contact with the police and judiciary system for so-called high impact crimes (43–45). Thus, we believe the results presented here highlight the importance of professionals in (mental) health and social care being actively involved in CVE initiatives. This is the best way to ensure that care is delivered to people with a complex need for care and thus it ensures a more integrated approach to violent extremism, in which a balance can be found between the objectives of community safety, case support and guidance (15). The measure of withdrawing a passport is illustrative of the need to find such a balance. This is possibly effective from a safety perspective, as it limits the possibilities for the person in question to travel abroad. At the same time, however, it is a problematic intervention from a (public) health perspective, as it may further limit a person's possibilities and self-sufficiency (e.g., obstructive in obtaining a health insurance or getting a job). Of course, this is true for any involvement with the police and the law; if a person has a judicial case pending, this is likely to interfere with any ongoing care, housing program or community-based activities (54). In many cases, judicial procedures will first have to be closed before other pathways can be started. As we saw in the current study, as much as 41.2% had self-sufficiency problems in the domain of Law and Order, meaning they had past or current contact with the courts. In an integrated CVE approach like the one in Amsterdam, the different individual risk and (lack of) protective factors are weighed and a shared decision is made about which measures will probably contribute most to prevention of further radicalization or extremist behavior. Or, on the other hand, which measures may cause harm and in fact increase the risk of violent behavior.

Mental health professionals may play an important role in continually stressing the complex relation between mental health, psychopathology and radicalization or terrorism. They can prevent the framing of mental health problems in a stigmatizing manner by analyzing individual cases and help to clarify whether treatment and care of mental health and self-sufficiency problems might contribute to deradicalization or not (7). They can help identify cases in which psychopathology and vulnerability in a broader sense might be ruled out. Finally, mental health professionals can provide support to partners from law enforcement by explaining how disturbed behavior in individual cases may be triggered, and how to deal with it subsequently. For example, by explaining to law enforcement that clients with multiple and complex vulnerabilities such as described here, will sometimes struggle to comply and participate in case-based interventions given the deficits in self-sufficiency (3). Together, this may help prevent violent escalation when an individual is going to be approached by partners from law enforcement. In that sense a longitudinal multi-agency approach in which data from police and security services are combined with information from health care is most effective in dealing with a radicalized population with mental and social problems.

## Data Availability Statement

The datasets presented in this article are not readily available because the data contain highly confidential information. Requests to access the datasets should be directed to chgrimbergen@ggd.amsterdam.nl.

## Author Contributions

CG conceptualized/designed the study and gathered data from patient files. TF prepared the encoded data and conducted the data analysis. CG and TF contributed to designing the semi-structured interview and to writing the manuscript equally. Both authors have read and approved the final manuscript.

## Conflict of Interest

The authors declare that the research was conducted in the absence of any commercial or financial relationships that could be construed as a potential conflict of interest.

## Publisher's Note

All claims expressed in this article are solely those of the authors and do not necessarily represent those of their affiliated organizations, or those of the publisher, the editors and the reviewers. Any product that may be evaluated in this article, or claim that may be made by its manufacturer, is not guaranteed or endorsed by the publisher.
